# Epitope targeting with self-assembled peptide vaccines

**DOI:** 10.1038/s41541-019-0125-5

**Published:** 2019-07-19

**Authors:** David F. Zeigler, Emily Gage, Richard Roque, Christopher H. Clegg

**Affiliations:** 1 TRIA Bioscience Corp, Suite 260, 1616 Eastlake Avenue East, Seattle, WA 98102 USA; 2 Present Address: MedImmune, One MedImmune Way, Gaithersburg, MD 20878 USA

**Keywords:** Biotechnology, Immunology

## Abstract

Nanoparticle-based delivery systems are being used to simplify and accelerate new vaccine development. Previously, we described the solid-phase synthesis of a 61-amino acid conjugate vaccine carrier comprising a α-helical domain followed by two universal T cell epitopes. Circular dichroism, analytical centrifugation, and dynamic light scattering indicate that this carrier forms coiled-coil nanoparticles. Here we expand the potential of this carrier by appending B cell epitopes to its amino acid sequence, thereby eliminating the need for traditional conjugation reactions. Peptides containing Tau or amyloid-β epitopes at either terminus assemble into ~20 nm particles and induce antibody responses in outbred mice. Vaccine function was verified in three experiments. The first targeted gonadotropin-releasing hormone, a 10-amino acid neuropeptide that regulates sexual development. Induction of peak antibody titers in male mice stimulated a dramatic loss in fertility and marked testis degeneration. The second experiment generated antibodies to an epitope on the murine IgE heavy chain analogous to human IgE sequence recognized by omalizumab, the first monoclonal antibody approved for the treatment of allergic asthma. Like omalizumab, the anti-IgE antibodies in immunized mice reduced the concentrations of circulating free IgE and prevented IgE-induced anaphylaxis. Finally, a peptide containing the highly conserved Helix A epitope within the influenza hemagglutinin stem domain induced antibodies that successfully protected mice against a lethal H1N1 challenge. These results establish the utility of a new vaccine platform for eliciting prophylactic and therapeutic antibodies to linear and helical B cell epitopes.

## Synthetic vaccines: Broad efficacy nanoparticle vaccine

Synthetic nanoparticles have the potential to be a simple, efficacious, and customizable platform for vaccine delivery. Christopher H. Clegg and colleagues include B cell epitopes on a self-assembling α-helical peptide nanoparticle carrier in order to elicit robust antibody generation. This novel vaccine platform is validated in vivo to produce physiologically-relevant antibodies in three different settings: an antibody-mediated ‘castration’ approach (anti-gonadotropin-releasing hormone), depletion of IgE (by generation of anti-IgE), and finally production of antibodies to a conserved H1N1 influenza epitope that mediates a protective effect in mice.

## Introduction

Nanoparticle-based vaccines increase immunogenicity by mimicking structural features of microbial pathogens (e.g., particle size, composition, valency, etc.) that stimulate adaptive immunity.^1–3^ For example, virus-like particle (VLP) vaccines use self-assembling capsid proteins to form an immunogenic scaffold, which can be modified with antigens. Other formats include liposomal, polymeric (e.g., PLA, PLGA, Chitosan), and inorganic (e.g., gold, ferritin, carbon) nanoparticles. A more recent technology utilizes self-assembling peptides that rely on protein oligomerization motifs to form immunogenic structures.^4,5^ The two most common examples comprise β-sheets of alternating hydrophilic and hydrophobic amino acids (AAs) that generate filamentous particles or α-helical peptides that use a repeating 7-AA heptad sequence to form coiled-coil assemblies.^6^

Conjugate vaccines require a protein carrier to increase the immunogenicity of weak antigens and are among the most successful vaccines developed to date.^7^ Previously, we described a 61-AA carrier (P8) that is manufactured by solid-phase peptide synthesis (SPPS) and contains 5 “IKKIEKR” heptads to mediate parallel trimeric coiled-coil assembly.^8^ The isoleucines interdigitate to form a hydrophobic core that is stabilized by salt bridges between glutamic acid and arginine^6^; the solvent-exposed lysines can be used for hapten conjugation. Downstream of these 5 heptads are CD4 T cell epitopes (TCEs) required for durable antibody (Ab) responses. Typically isolated from common pathogens (i.e., tetanus, measles, hepatitis B), these peptide epitopes bind a broad repertoire of major histocompatibility complex class II alleles in rodents, pigs, monkeys, and humans.^9–12^ We have used this carrier to produce a nicotine vaccine for smoking cessation and have shown that the Ab concentrations induced in mice and rats can bind supraphysiological doses of nicotine and prevent nicotine-induced toxicity.^8,13,14^

The only Food and Drug Administration-approved conjugate vaccines prevent microbial infection via serotype-specific polysaccharides linked to carrier proteins.^7^ There have been numerous clinical attempts to elicit therapeutic Ab levels to peptide B cell epitopes (BCEs) to treat other conditions, such as cancer,^15^ Alzheimer’s disease,^16^ and allergy.^17,18^ Here we present a simple paradigm for eliciting immune responses to these BCEs by appending them to the termini of our carrier during SPPS. This method eliminates conventional conjugation reactions, which can negatively impact vaccine activity. These peptides are easily purified, self-assemble into thermostable ~20–30 nm particles, and induce strong functional Ab responses in mice.

## Results

**Fig. 1:**
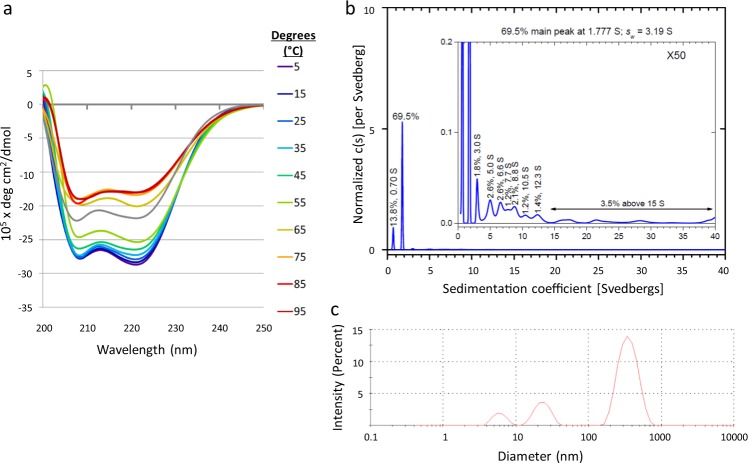
P8 peptide forms a heat stable trimeric coiled-coil. **a** Circular dichroism spectra: P8 was dissolved in phosphate-buffered saline and subjected to increasing temperature from 5 °C to 95 °C, followed by a return to 5 °C (gray line). **b** Analytical ultracentrifugation spectrum of P8 showing monomer (0.76 S), trimer (1.78 S), and higher-order assemblies (>3 S). **c** Intensity-weighted dynamic light scattering spectrum showing major self-assembly (7 nm) and two minor assemblies (25 and 350 nm)

The P8 carrier peptide used in our initial nicotine vaccine experiments has a molecular weight of 7.38 kD and contains a 5 heptad repeat sequence followed by PADRE and influenza-derived TCEs (Table S1).^8^ P8 was characterized by circular dichroism (CD), analytical ultracentrifugation (AUC), and dynamic light scattering (DLS). The minima at 208 and 222 nm in the CD plot (Fig. 1a) delineate a clear α helical signature, and as indicated by 222/208 nm ratios >1, the peptides maintain a stable coiled-coil conformation up to 65 °C.^19,20^ The size distribution of the carrier was then determined by AUC (Fig. 1b). Three main species sedimented at 0.76, 1.77, and >3.0+ Svedberg (S) units, corresponding to peptide assemblies of 14% monomers (~7.5 kDa), 70% trimers (~22 kDa), and 16% higher-order assemblies (hexamers, nanomers, dodecamers, etc.). When analyzed by DLS, P8’s intensity spectrum exhibits three peaks at approximately 7, 22, and 350 nm (Fig. 1c). The areas under these peaks are weighted based on the relative size of the species; larger species scatter more photons and lead to disproportionately higher intensities. After the intensity spectrum is un-weighted to a number spectrum, the 7-nm assembly accounts for most of the mixture and corresponds to the trimeric coiled-coil (1.77 S). This implies that the 22-nm peak represents the 3–15 S assemblies, and the 350 nm peak contains larger aggregates unmeasurable by AUC. Thus the P8 carrier has α-helical secondary structure and assembles primarily as a stable trimeric coiled-coil.

**Fig. 2:**
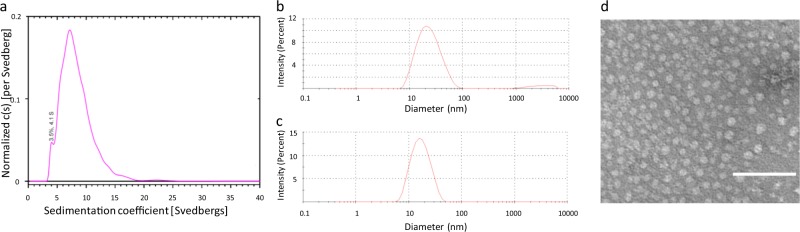
P10 and P15 self-assembled nanoparticles. **a** Analytical ultracentrifugation spectrum of P10 showing a major peak at 7 S. **b** Dynamic light scattering (DLS) spectrum of P10 showing ~20 nm assemblies. **c** DLS spectrum of P15 peptide showing ~15–20 nm assemblies. **d** Transmission electron microscopic (TEM) micrograph of P10 showing similar assembly sizes. TEM scale bar is 100 nm

In addition to P8, we have tested the carrier activity of two peptides containing different universal TCEs: P10 contains epitopes isolated from measles virus fusion 2 and Hepatitis B surface antigen proteins and P15 contains three overlapping TCEs isolated from tetanus toxoid protein (Supplementary Table 1).^14^ However, unlike P8, no trimer peaks were detected for these peptides by AUC and DLS (Fig. 2a–c). Thus we conclude that the IKKIEKR heptad repeats in these peptides mediate self-assembly into coiled-coils but that AAs within the TCE domain can influence formation of higher-order assemblies. Transmission electron microscopy (TEM) of P10 revealed circular assemblies of approximately 10–20 nm in size, consistent with its un-weighted DLS profile (Fig. 2d). Based on the quantitative agreement between these biophysical measurements, DLS was used as a high-throughput method to verify nanoparticle formation.

Conjugate vaccines are being tested in the clinic for their ability to induce therapeutic Ab responses. Vaccines for Alzheimer’s disease have focused on coupling short synthetic peptides found in the plaque-forming N-terminus of amyloid-β to various carriers including KLH, CRM_197_, and Qb phage VLPs.^16^ Another Alzheimer’s vaccine uses KLH conjugated to a 12-AA peptide within Tau, the protein found in neurofibrillary tangles.^21^ To test the immunogenicity of carrier peptides synthesized with these very same BCEs, we shortened P15’s length by removing a heptad repeat and placed amyloid-β (EFRHDSGY) or Tau (KDNIKHVPGGGS) epitopes at either terminus, after which they were formulated with GLA-SE adjuvant^22^ and injected into outbred CD-1 mice. DLS analysis established that the nanoparticle sizes of these modified peptides were similar to P15 (Supplementary Fig. 1), and as shown in Supplementary Fig. 2, both pairs of amyloid-β and Tau peptides successfully induced strong Ab titers. No significant differences in response were seen when the epitope was placed at either end of the peptide.

**Fig. 3:**
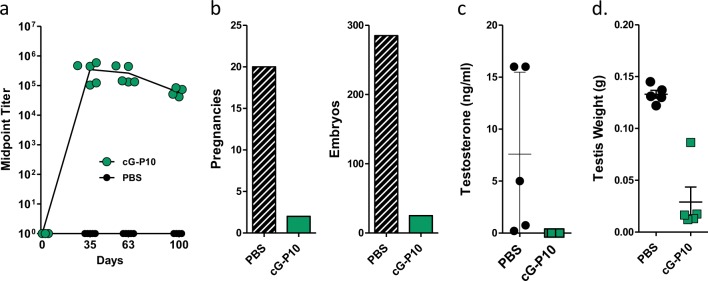
Peptides targeting gonadotropin-releasing hormone (GnRH) induce an immunocastration phenotype in mice. The 10 amino acid GnRH sequence was synthesized onto the C-terminus of P10 (cG-P10). CD-1 male mice (5/group) were immunized (d0, d21, d42) with phosphate-buffered saline or 10 µg of peptide and GLA-SE and then assayed for: **a** anti-GnRH antibody titers at designated time points, **b** fertility based on the number of pregnancies and embryos resulting from breeding immunized male mice with 4 female mice between d40 and d80, **c** testosterone levels in d63 serum, and **d** testis weights on d100. Mouse immunization studies were performed in triplicate; testosterone and fertility analysis was performed once. Error bars represent mean ± SD

To explore the therapeutic activity of this vaccine platform, we focused on gonadotropin-releasing hormone (GnRH), a 10-AA neuropeptide that regulates sexual development and function. Several GnRH vaccines using different carriers are being marketed as immunocastration vaccines for animal reproduction and husbandry purposes.^23,24^ In a preliminary experiment, we established that a C-terminal murine GnRH sequence (QHWSYGLRPG) on P10 and P15 formed nanoparticle sizes comparable to their unmodified carriers and that cGnRH-P10 peptide induced substantially better Ab responses than a P15-based vaccine (Supplementary Fig. 3). Owing to P10’s superior immunogenicity, we tested cGnRH-P10 for its ability to castrate male CD-1 mice and P10 was used as the carrier for future experiments. Following immunization, anti-GnRH Ab titers reached maximal levels after the first boost and remained high throughout the course of the experiment. To confirm that these Abs could inhibit GnRH, we bred these males with four females and measured their fertility (Fig. 3b). All 5 control mice impregnated each female successfully and collectively produced 285 embryos. In contrast, no pregnancies occurred in 4 of the 5 immunized mice and the fifth mouse impregnated just 2 of the 4 females, resulting in a total of 25 embryos. Additional proof that these GnRH Abs effectively bind and neutralize the endogenous hormone was corroborated by undetectable testosterone levels in day 63 sera (Fig. 3c), a ten-fold reduction in testis weight (Fig. 3d), and a testis architecture with severe tubular degeneration (Supplementary Fig. 4).

**Fig. 4:**
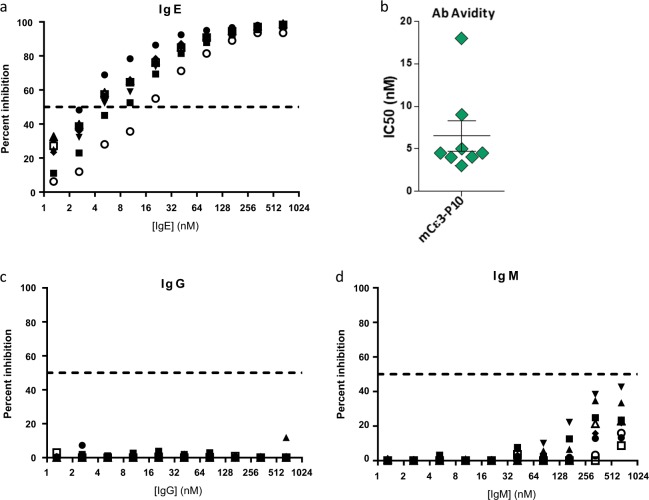
P10 was synthesized with the murine Cε3 sequence corresponding to the human epitope recognized by omalizumab. **a**, **b** Competition enzyme-linked immunosorbent assay showing that the antibodies induced in d35 antisera bound specifically to mouse IgE with nanomolar affinities, whereas the same antisera failed to bind mouse IgG (**c**) and IgM (**d**). The different symbols used in **a**, **c**, and **d** represent antisera from individual animals in each group (*n* = 8). Mouse immunization studies were performed in duplicate. Error bars represent mean ± SD

**Fig. 5:**
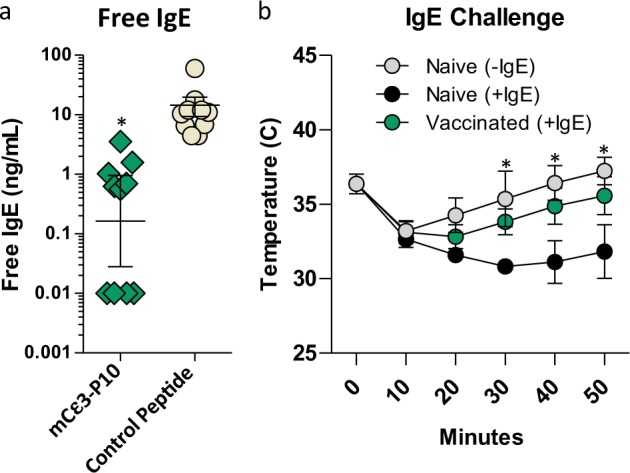
Inhibition of free-IgE and systemic anaphylaxis in immunized mice. **a** Day 35 antisera from immunized (*n* = 10) and control (*n* = 10) animals were assayed for free IgE in a competition enzyme-linked immunosorbent assay that measures the amount of IgE available for binding to the mFceRI receptor through the Cε3 loop (unpaired *t* test, *p < 0.002). **b** Vaccinated (*n* = 4) and naive mice (*n* = 4) were anesthetized and injected (intraorbital) with 0.5 µg of DNP-specific IgE and then DNP-HAS 24 h later. Anaphylaxis was measured by a reduction in body temperature. Anesthesia alone caused a drop in temperature as in naive mice not injected with IgE (gray circles). No significant difference in body temperatures were observed between the vaccinated and naive (−IgE) mice except at 20 min (*p* < 0.05). Differences in body temperatures between naive (+IgE) and vaccinated (+IgE) were significantly different after the first 10 min (one-way analysis of variance; *p* < 0.05). Anaphylaxis experiment performed as a single replicate. Error bars represent mean ± SD

To corroborate the functional utility of this vaccine design, we tested the concept of an anti-IgE vaccine for treating allergic hypersensitivities. The first monoclonal Ab (mAb) approved for allergic asthma, omalizumab, recognizes a linear epitope on the Cε3 loop within the IgE heavy chain and prevents IgE receptor activation.^25,26^ P10 peptide synthesized with the murine homolog of omalizumab’s epitope (DHPDFPKPIV)^18,26^ at its C-terminus exhibited an average DLS diameter of ~15 nm (Supplementary Fig. 5). Antisera from immunized mice specifically bound murine IgE with nanomolar avidity but not murine IgG or IgM (Fig. 4), and like omalizumab, these anti-IgE Abs were able to reduce the concentration of free IgE ~10-fold in 6 of the 10 mice and ~1000-fold in 4 of the 10 mice (Fig. 5a). Importantly, this vaccine could also be used to inhibit acute IgE-mediated anaphylaxis, confirming its therapeutic potential (Fig. 5b).

**Fig. 6:**
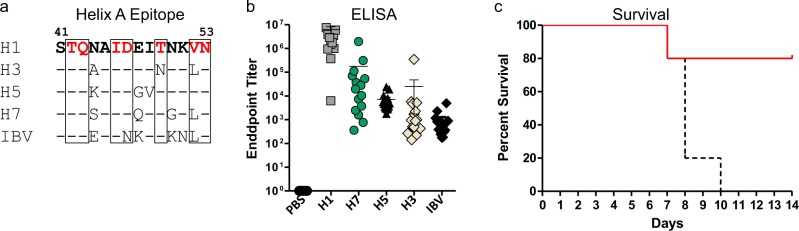
Peptides modified with the Helix A (HxA) epitope protect mice from lethal virus challenge. **a** HxA epitopes from four strains of influenza A and one strain of influenza B viruses. The position of the epitope within the HA2 domain of hemagglutinin (HA) is denoted by the residue numbers shown above the sequence. The CR9114 monoclonal antibody contact residues are identified by the red amino acids and boxes. Conserved residues are indicated by a dash. **b** The HxA epitope from A/California/07/2009 (H1N1) was appended to the N-terminus of the alpha-helical domain of P10. CD-1 male mice (15/group) were immunized (d0, d21) with 10 µg of peptide plus adjuvant and d35 antisera were assayed for cross-reactive binding to the indicated HA antigens by enzyme-linked immunosorbent assay. Error bars represent mean ± SD. **c** Mice (*n* = 5) immunized with phosphate-buffered saline (dashed black line) and HxA^H1^-P10 (solid red line) were challenged on day 40 with 10 × LD_50_ of A/California/07/2009 and monitored for survival with a weight loss cutoff of 75%. Mouse challenge studies were performed in triplicate

Finally, we tested whether this platform could be used to protect against influenza virus, whose diversity and rapid antigenic drift has hampered the development of an effective vaccine. mAbs that neutralize a broad range of viruses have helped identify potential epitopes for an improved vaccine.^27^ For instance, CR9114 binds a conformational epitope within the hemagglutinin (HA) stem domain that encompasses a hydrophobic pocket and the outward facing residues of the neighboring Helix A (HxA) (Fig. 6a).^28^ The CR9114 contact residues are remarkably well conserved between Group 1 and Group 2 influenza A viruses and B viruses and the few differences that do exist represent relatively conserved substitutions. Such non-linear epitopes are difficult to recreate in conjugate vaccines. However, we hypothesized that the α-helical heptad domain of our carrier could be used to constrain the HxA primary sequence to its native helical state with the CR9114 contact residues facing outward (Supplementary Fig. 6). The HxA sequence from H1N1 was included at the N-terminus of P10 (HxA^H1^-P10); this antigen exhibited a slightly larger diameter (~27 nm) than the previous peptides, presumably due to its extended coiled-coil structure. Antisera from mice immunized with HxA^H1^-P10 showed strong binding to recombinant H1 and significant cross-reactive binding to other HA proteins (Fig. 6b). Single substitutions in the CR9114 contact residues in H7 and H5 decreased anti-sera binding, while double substitutions in H3 and IBV resulted in proportionally weaker cross-reactivity.^29^ Further analysis established that the vaccine induced a Th1 CD4 T cell response, which is a property of TLR4 activation by GLA adjuvant (Supplementary Fig. 7).^22^ Importantly, mice immunized with HxA^H1^-P10 were protected against a 10xLD_50_ challenge dose with H1N1 virus (Fig. 6c), thus demonstrating that this new vaccine platform can be used to induce protective antiviral responses to conformational α-helical epitopes.

## Discussion

An important public health goal is the manufacture of safe and effective vaccines that are inexpensive and easily distributed worldwide. One innovation that simplifies vaccine development is the use of self-assembled nanoparticles, which display a repetitive antigen structure critical for B cell activation and a size that facilitates antigen processing and transport within lymphoid cells and tissues. While several of these platforms are being developed, one of the most common relies on α-helical peptide motifs. For instance, Burkhard’s group has built functional vaccines for both malaria and influenza that combine pentameric and trimeric coiled-coil oligomerization domains with appropriate BCE and TCE combinations.^30,31^ Here we present a vaccine platform based on an alternative α-helical motif that can elicit therapeutic and prophylactic Ab responses to a variety of linear and helical BCEs.

Previously, we reported the in vivo activity of several peptide carriers with different TCE combinations for smoking cessation vaccines; the peptides used in these studies (P8, P10, and P15) were subjected to a standard battery of physical characterization techniques.^14^ We confirmed by CD analysis that P8 has an α-helical secondary structure and assumes a stable coiled-coil assembly based on the 222/208 nm peak ratio.^19,20^ However, above 65 °C, the coils undergo a conformational change that could reflect fraying of the non-heptad TCE terminus or other disruptions of the coiled-coil assembly. AUC indicated that 70% of the peptides exist as trimeric assemblies, with the remaining 30% comprising monomers and higher-order assemblies. DLS corroborated the presence of these species, plus a small amount of very large aggregates undetectable by AUC. In contrast to P8, no trimer was detected in P10 and P15 peptides; these assemblies had sizes comparable to the intermediate P8 nanoparticles (~20 nm), showing that TCE sequences influence self-assembly thermodynamics and nanoparticle size.

To test whether these peptides could be used for epitope targeting, we selected short peptide sequences from amyloid-β and Tau proteins, both of which have been used in clinical-stage conjugate vaccines for Alzheimer’s disease.^16,21^ P15 peptides synthesized with N- or C-terminal epitopes showed the same general distribution in particle size, although N-terminal additions were slightly larger. This may reflect how the N-terminal BCE projects from the nanoparticle surface, creating a larger DLS diameter. It may also suggest that flanking the heptad repeat with relatively hydrophobic BCE and TCE sequences can influence inter-peptide interactions and resultant assembly size and shape. Importantly, both the Tau and amyloid-β epitopes induced strong Ab responses in outbred mice at either terminus. Thus the Ab epitopes presented by these nanoparticles can readily engage and activate B cells.

To further investigate the therapeutic utility of these synthetic vaccines, we targeted self-epitopes that would induce unambiguous phenotypes in mice. For instance, GnRH plays a prominent role in regulating sexual development and function. Produced within the hypothalamus, this neuropeptide stimulates the synthesis and release of follicle-stimulating hormone and luteinizing hormone from the pituitary, which in turn control androgen and estrogen production. GnRH conjugate vaccines that employ KLH and diphtheria toxoid carriers are being used to inhibit reproduction of domestic and wild animals, controlling meat quality in pigs and reducing aggressive behaviors in livestock.^23,24^ Here we established that peptides synthesized with the murine GnRH sequence induced strong anti-GnRH Ab responses and that these Abs could mediate a dramatic loss in fertility and normal testes morphology. In addition, the P10 vaccine showed enhanced immunogenicity relative to its P15 analog, which is consistent with our anti-nicotine vaccines experiments.^14^ Future experiments will determine whether the elevated potency of P10 TCEs is retained in larger animals. A second therapeutic experiment targeted the mouse IgE heavy chain epitope analogous to omalizumab’s IgE-binding site.^18,25,26,32^ Competition enzyme-linked immunosorbent assays (ELISAs) established that the Abs induced in mice specifically bound IgE with nanomolar avidity and reduced the concentrations of circulating free IgE. Moreover, this same Ab response effectively suppressed an IgE-mediated anaphylactic response. These experiments suggest that this vaccine platform might prove useful for targeting peptide epitopes in therapeutic settings, such as allergy, cancer, hypertension, and animal health.^33–35^

In addition to these indications, this approach can also be used to prevent infectious disease. One strategy for building a universal influenza vaccine focuses on targeting highly conserved surface epitopes shared by different strains of virus.^36,37^ An epitope recognized by several broadly neutralizing mAbs (e.g., CR9114) includes HxA, the first alpha helical domain within the HA stalk.^27,28^ Here we successfully recreated the secondary structure of the HxA epitope by appending the linear HxA H1N1 sequence on the helical N-terminus of the carrier. In silico modeling affirms that correct positioning of CR9114 contact residues in outward-facing heptad positions ensures proper presentation of the secondary structure of the epitope. Mice induced cross-reactive Abs to drifted strains of type A and B viruses and were protected from a lethal (10 × LD_50_) H1N1 viral challenge. This contrasts with previous efforts to target this same epitope in its native conformation using an icosahedral VLP.^38^ Collectively, our results show the potential for simultaneously targeting conserved linear and α-helical domains on influenza viruses and other pathogens. Recently, we confirmed that these peptides can be formulated in multivalent vaccines for improved efficacy, which could be useful for targeting several infectious disease epitopes concurrently.^14^

Despite their success, conjugate vaccines are expensive to develop and the carrier–hapten coupling reaction can lead to diminished activity via perturbations in carrier structure, variable hapten/peptide loadings, accumulation of small molecular weight adducts, and aggregation.^39–41^ Consequently, their manufacturing must be carefully controlled and requires thorough processing and purification methodologies. However, the production of these peptide vaccines by SPPS eliminates conjugation chemistries and the potential for diminished activity, and it greatly simplifies manufacturing and development requirements. Evidence suggesting that this is a superior approach is based on the observation that inclusion of nicotine haptens during SPPS enhances vaccine activity relative to peptides conjugated to nicotine using traditional methodology.^14^ Another concern with conjugate vaccines is that their recombinant carriers are strongly immunogenic and induce anti-carrier Abs that can suppress vaccine activity.^41^ However, the small size and minimal sequence complexity of these peptides reduce anti-carrier responses substantially and are orders of magnitude lower than those induced by the commercial carrier CRM197.^14^ The simplicity and potency of this class of peptides suggests that they may be a promising tool for accelerating antigen discovery and new vaccine development.

## Methods

This study was carried out in strict accordance with the recommendations in the Guide for the Care and Use of Laboratory Animals of the National Institutes of Health, the US Public Health Service Policy on Humane Care and Use of Laboratory Animals, and the Association for Assessment and Accreditation of Laboratory Animal Care International (AAALAC). Protocol #2015-11 was approved by the Institutional Animal Care and Use Committees of the Infectious Disease Research Institute, which operates under a currently approved Assurance #A4337-01 and USDA certificate #91-R-0061.

### Peptides

All peptides were synthesized at Bio-Synthesis Inc. (Lewiston, TX). The P8 carrier peptide used in our previous experiments contains five IKKIEKR heptad repeats in the coiled-coil domain followed by the PADRE TCE (AKFVAAWTLKAAA) and a TCE isolated from Influenza H5N1 HA (YQNPTTYISVK).^8^ The P10 carrier peptide contains the same five heptad repeats followed by a TCE selected from Measles virus F2 protein (LSEIKGVIVHRLEGV) and Hepatitis B surface antigens (FFLLTRILTIPQSLD).^11^ The P15 carrier peptide contained three overlapping TCEs isolated from tetanus toxoid (FNNFTVSFWLRVPKVSASHLEQY).^12^ All peptides synthesized with linear BCEs had four IKKIEKR heptad sequences and two TCEs (Fig. 1). The amyloid-β BCE (EFRHDSGY) was synthesized at the N- and C-termini of P15 with Gly-Gly-Pro or Pro-Gly-Gly linkers, respectively. The Tau epitope (KDNIKHVPGGGS) was synthesized at the N- and C-termini of P15 with a Pro linker. The GnRH epitope (QHWSYGLRPG) was synthesized at the C-terminus of P10 with a Gly linker. The murine Cε3 epitope (DHPDFPKPIV)^32^ was synthesized at the C-terminus of P10 with a Gly linker. The HxA epitope (STQNAIDEITNKVN)^28^ was synthesized at the N-terminus of P10.

Peptide stock solutions were prepared for CD spectroscopy using 50 mM phosphate-buffered saline (PBS). Spectra were recorded from 190 to 270 nm on a Jasco J720 spectropolarimeter (Easton, MD) using 10 mm path length cells. Temperatures ranged from 5 to 95 °C in increments of 10 °C. Analytical ultracentrifugation was performed by Alliance Protein Laboratories (San Diego, CA). P8 was dissolved in PBS to ~1 mg/mL and P10 was dissolved in aqueous MOPS (100 mM, 50 mM NaCl, pH 7.5) to ~0.5 mg/mL. Samples were filtered through a 0.2-μm nylon membrane. The samples were loaded into a Beckman-Coulter ProteomeLab XL-A analytical ultracentrifuge (Brea, CA). After equilibration at 20 °C, the rotor was brought to 60,000 rpm. Scans were recorded every 4 min for ~10 h. The data were analyzed using SEDFIT (version 11.3).^42^ The resultant size distributions were graphed, and the peaks were integrated using OriginLab Origin® version 9.0 (Northampton, MA). DLS spectroscopy was performed using a Zetasizer Nano (Malvern Instruments, UK) with a 4 mW He–Ne laser (633 nm) and a fixed detection angle (173°). Prior to measurement, peptides were raised in PBS or MOPS (100 mM, 50 mM NaCl, pH 7.5), filtered through a 0.2-μm nylon membrane, and loaded into a plastic microcuvette. Measurements were carried out in general purpose model with the following parameters: material setting was protein (refractive index = 1.440), dispersant setting was water (viscosity = 0.8872 cP, refractive index = 1.330), 10 cycles averaged per measurement, and 30 s temperature equilibration at 25 °C. For transmission electron microscopy, peptides (0.05 mg/mL in PBS) were adsorbed onto carbon-coated grids, washed, and negatively stained with 0.5% uranyl acetate (Charles River, Durham, NC). Electron micrographs were taken on a JEOL 1400 Transmission Electron Microscope.

### Peptide modeling

The P10 structure was constructed, analyzed, and rendered with Chimera software^43^ using the trimeric coiled-coil construct without TCEs as a template. P10 TCEs were modeled using the PEP-FOLD3^44^ server and coupled to the trimeric coiled-coil carrier at the C-terminus using the structure building tools in Chimera to form peptide bonds. The Qwik-MD tool in VMD^45^ was used for molecular dynamics set-up, solvation/ionization, and a 1000-step equilibration.

### Animals

CD-1 mice (Charles River Laboratories) were housed under pathogen-free conditions in the Infectious Disease Research Institute vivarium (Seattle, WA). Peptide vaccines were dissolved in PBS and final concentrations were determined by AA analysis (AAA Service Laboratory, Damascus, OR). The peptides were combined on the day of immunization with GLA-SE adjuvant containing 5 µg of the synthetic TLR4 agonist, GLA, formulated in a final 2% oil-in-water stable emulsion.^22,46^ The adjuvant was provided by Immune Design Corp (Seattle, WA). Each mouse received 10 µg peptide diluted in 100 µL total volume, 50 µL of which was injected into each hind limb on days 0, 21, and 42. Serum was collected and used to measure Ab responses. To measure fertility, each CD-1 male was housed with 4 females between day 40 and day 80 post-immunization. Pregnant females were sacrificed and embryos (d9–d14) were counted. Testis weights were measured 100 days after the prime. Tissues were stored in 10% neutral buffered formalin and then processed for hematoxylin and eosin staining and histological analysis (Comparative Pathology Program, Seattle, WA). The mouse anaphylaxis model^47^ was performed as follows: immunized and control mice (5/group) were anesthetized with isoflurane and injected (intraorbital) with 0.25 µg DNP-IgE (Pharmingen; San Diego, CA). Twenty-four hours later, mice were then challenged by a second intraorbital injection with 1 mg of the antigen DNP-HSA. Anaphylaxis onset and duration was assessed by measuring body temperature at 10-min intervals using a rectal probe (Kent Scientific, Torrington, CT). Unchallenged naive mice were used to control for the effects of anesthesia on body temperature. Plotted data report the means ± SD and statistical differences between groups were determined by unpaired *t* test or one-way analysis of variance (*p* < 0.05). Influenza challenge experiments were performed by infecting mice intranasally with 10 × LD_50_ dose of A/CA/07/09 in 50 µL PBS. Mice were monitored for weight loss and other signs of virus-induced morbidity daily and sacrificed if weight loss exceeded 25% of the initial body weight.

### Antibody assays

Serum samples were serially diluted 3-fold from 1/100 in blocking buffer (3% bovine serum albumin (BSA) in PBST) and assayed by ELISA using standard methodologies. Midpoint titers at half maximal absorbance were calculated using GraphPad Prism (GraphPad Software, San Diego, CA). Coating concentration of proteins or Abs was 1 µg/mL. Amyloid-β and GnRH Abs were measured using cysteine-terminated synthetic peptides conjugated onto BSA using maleimide crosslinking chemistry. Tau Abs were measured using a 441-AA recombinant isoform of human Tau protein (rPeptide, Watkinsville, GA). Mouse testosterone was detected with a commercial kit purchased from Abcam (Cambridge, MA). IgE Ab titers were measured using mouse IgE (Biolegend, catalog #401701) as a coating reagent and goat anti-mouse IgG Fc-HRP (Southern Biotech, Birmingham, AL, catalog #1033-05) as the secondary at a dilution of 1:1000. Specificity of Ab binding and relative avidity to IgE was measured by competition ELISA.^48^ Serial dilutions of murine IgE, IgG (Thermo Fisher, catalog #31903), or IgM (Southern Biotech, catalog #0101-01) were preincubated with antisera for 1 h and then added to wells coated with these Ab isotypes for an additional hour. Goat anti-mouse IgG Fc-HRP was used for detection at a dilution of 1:16,000. ELISA signals were fit with an inhibition regression algorithm and IC_50_s determined for each group using GraphPad Prism. Free IgE in mice, or the amount of IgE unbound by anti-IgE Abs in serum, is measured by ELISA for its ability to bind receptor, where mFcεRI (NBS-C Bioscience, Austria) and goat anti-mIgE (Southern Biotech, Birmingham, AL, used at 1:8000 dilution) are the capture and detection reagents, respectively.^49^ HxA titers were measured using recombinant HA from H1N1 A/California/07/2009, H3N2 A/Wisconsin/67/2005, H5N1 A/Vietnam/1203/2004, and H7N9 A/Anhui/1/2013 or B/Malaysia/2506/2004 (Protein Sciences).

### T cell ELISPOT

Interferon-γ (R&D Systems, Minneapolis, MN, USA) ELISPOT analyses were conducted according to the manufacturer’s instructions. Briefly, splenocytes from immunized and unimmunized mice were resuspended and serially diluted in RPMI media supplemented with 10% fetal bovine serum and L-glutamine. Resuspended splenocytes were stimulated with 20 µg/mL P10-dimer, media alone, or phorbol 12-myristate 13-acetate and ionomycin for 48 h prior to development. Spot images were collected using ImmunoCapture 6.4 and analyzed with ImmunoSpot 5.0 on an automated ELISPOT plate reader (C.T.L. Seri3A Analyzer; Cellular Technology, Shaker Heights, OH, USA).

### Reporting summary

Further information on research design is available in the Nature Research Reporting Summary linked to this article.

## Supplementary information

Supplementary Figures

Reporting Summary

## Data Availability

The data that support the findings of this study are available from the corresponding author upon reasonable request.

## Supplementary information

**Supplementary information** accompanies the paper on the *npj Vaccines* website (10.1038/s41541-019-0125-5).
