# Supplementary material for: Epitope targeting with self-assembled peptide vaccines
Source: NPJ Vaccines. 2019 Jul 19;4:30. doi: 10.1038/s41541-019-0125-5 (PMC6642127; doi:10.1038/s41541-019-0125-5)
Supplement: Supplementary file 1 — Supplementary Figures [file 41541_2019_125_MOESM1_ESM.pdf]

Supplementary Table 1. Peptide Vaccine Sequences

| Peptide name | N-terminal B cell epitope | Heptad domain sequence | T cell epitope domain sequence | C-terminal B cell epitope |
|---|---|---|---|---|
| P8 | | DKIEKRIKKIEKRIKKIEKRIKKIEKRIKKIEKRIKK | AKFVAAWTLKAAAYQNPTTYISVK | |
| P10 | | DKIEKRIKKIEKRIKKIEKRIKKIEKRIKKIEKRIKKWWP | LSEIKGVIVHRLEGFFLLTRILTIPQSLD | |
| P15 | | DKIEKRIKKIEKRIKKIEKRIKKIEKRIKKIEKRIKKWP | FNNFTVSFWLRVPKVSASHLEQY | |
| nTau-P15 | KDNIKHVPGGGSP | DKIEKRIKKIEKRIKKIEKRIKKIEKRIKKWP | FNNFTVSFWLRVPKVSASHLEQY | |
| cTau-P15 | | DKIEKRIKKIEKRIKKIEKRIKKIEKRIKKWP | FNNFTVSFWLRVPKVSASHLEQY | KDNIKHVPGGGS |
| nAbeta-P15 | EFRHDSGYGGP | DKIEKRIKKIEKRIKKIEKRIKKIEKRIKKWP | FNNFTVSFWLRVPKVSASHLEQY | |
| cAbeta-P15 | | DKIEKRIKKIEKRIKKIEKRIKKIEKRIKKWP | FNNFTVSFWLRVPKVSASHLEQY | PGGEFRHDSGY |
| cGnRH-P10 | | DKIEKRIKKIEKRIKKIEKRIKKIEKRIKKWWP | LSEIKGVIVHRLEGVFFLLTRILTIPQSLD | GQHWSYGLRPG |
| cGnRH-P15 | | DKIEKRIKKIEKRIKKIEKRIKKIEKRIKKWP | FNNFTVSFWLRVPKVSASHLEQY | GQHWSYGLRPG |
| mCε3-P10 | | DKIEKRIKKIEKRIKKIEKRIKKIEKRIKK | LSEIKGVIVHRLEGVFFLLTRILTIPQSLD | GHPDFPKPIVRS |
| $HxA^{H1}$-P10 | STQNAIDEITNKVN | IKKIEKRIKKIEKRIKKIEKRIKKIEKRIKK | LSEIKGVIVHRLEGVFFLLTRILTIPQSLD | |
| $HxA^{H1}$-P15 | STQNAIDEITNKVN | IKKIEKRIKKIEKRIKKIEKRIKKIEKRIKK | FNNFTVSFWLRVPKVSASHLEQY | |

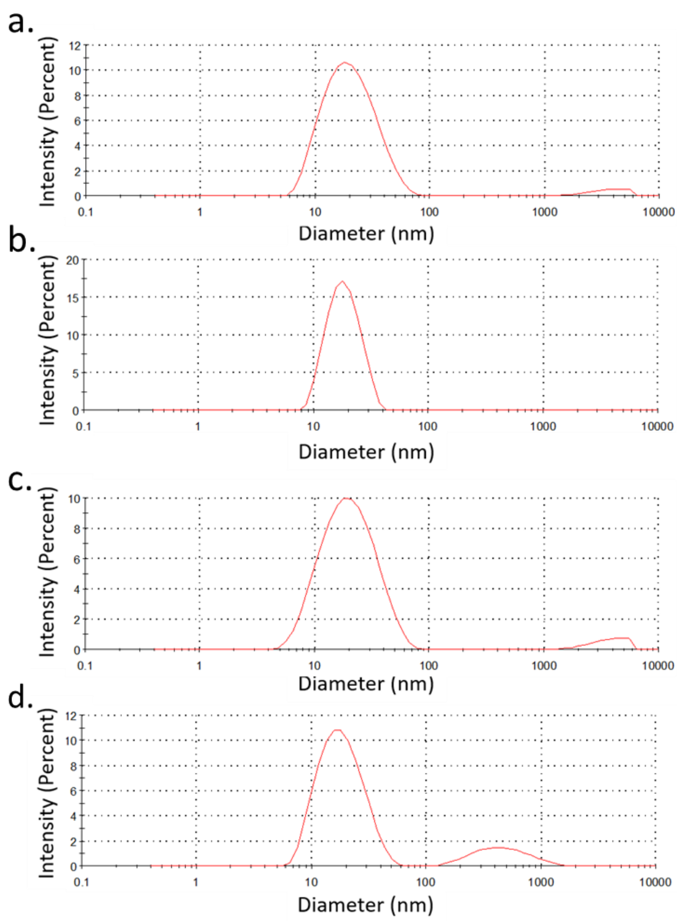


**Supplementary Figure 1.** Dynamic light scattering analysis of peptides containing tau and amyloid-beta epitopes incorporated at the N- or C-terminus of the carrier peptide. **(a)** nTau-P15, **(b)** cTau-P15, **(c)** nAβ-P15, and **(d)** cAβ-P15

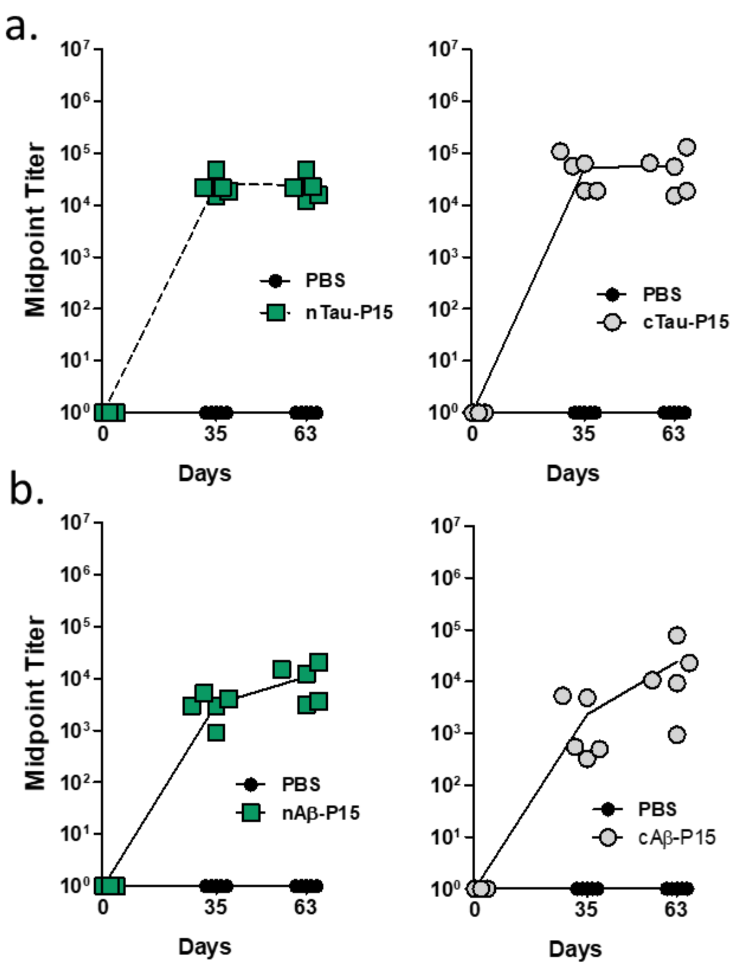


**Supplementary Figure 2.** Antibody responses from peptides modified with tau and amyloid-b epitopes. BCEs were synthesized onto the N- or C-terminus of P15. CD-1 female mice (n=5) were immunized on days 0, 21 and 42 with 10 µg of peptide adjuvanted with GLA-SE. Serum was collected on day 56 and titers assayed by ELISA. **(a)** nTau-P15 and cTau-P15; **(b)** nAβ-P15 and cAβ-P15. Mouse immunization studies were performed once.

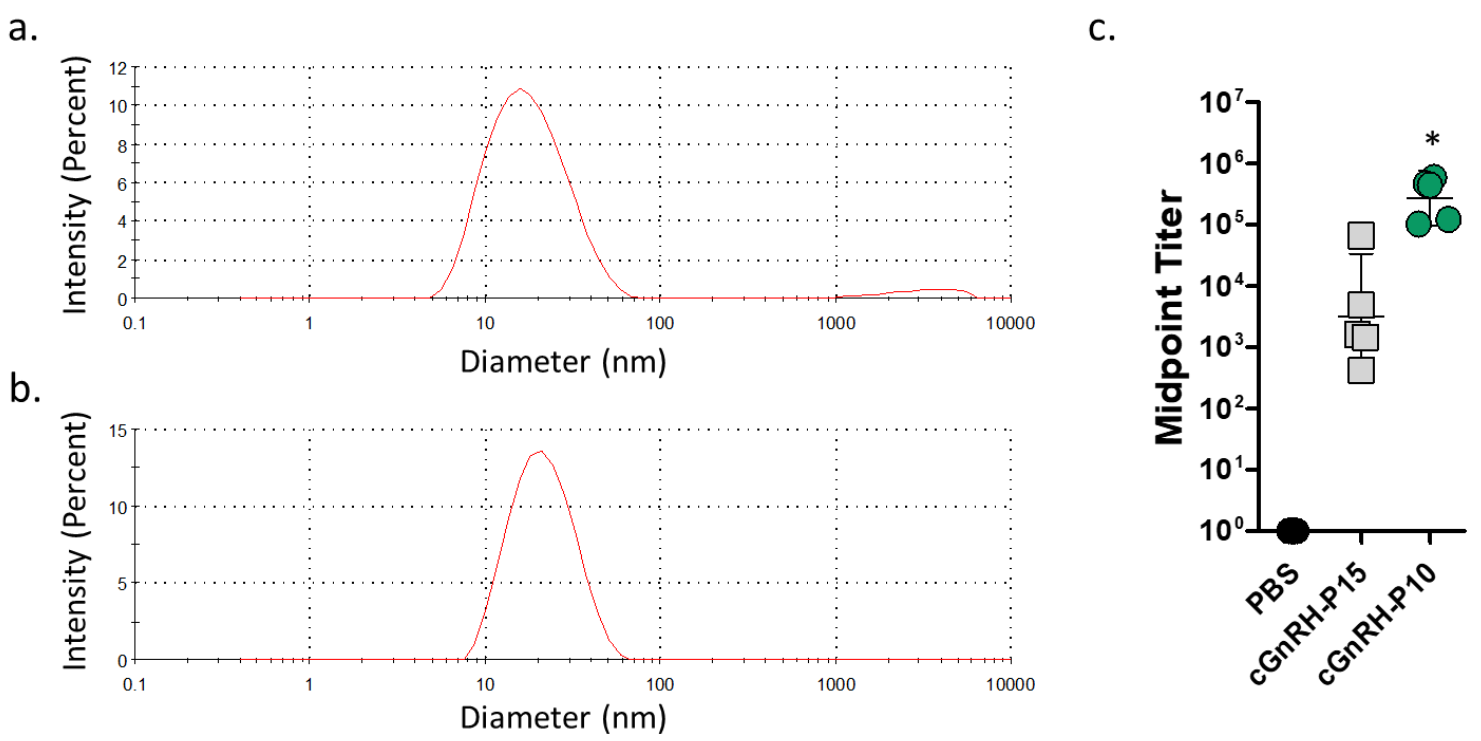


**Supplementary Figure 3.** TCE domains influence antibody responses in immunized mice. P15 and P10 peptides were synthesized with a C-terminal GnRH epitope (Supplemental Table 1). **(a)** DLS analysis of cGnRH-P15. **(b)** DLS analysis of cGnRH-P10. **(c)** CD-1 male mice (5/grp) were immunized (d0, d21) with 10 µg of peptide and adjuvant and d35 antisera were assayed by ELISA. Statistical difference between groups is indicated by the asterisk (unpaired t-test; *$p<0.011$). Error bars represent mean $\pm$ SD. Mouse immunization studies were performed in duplicate.

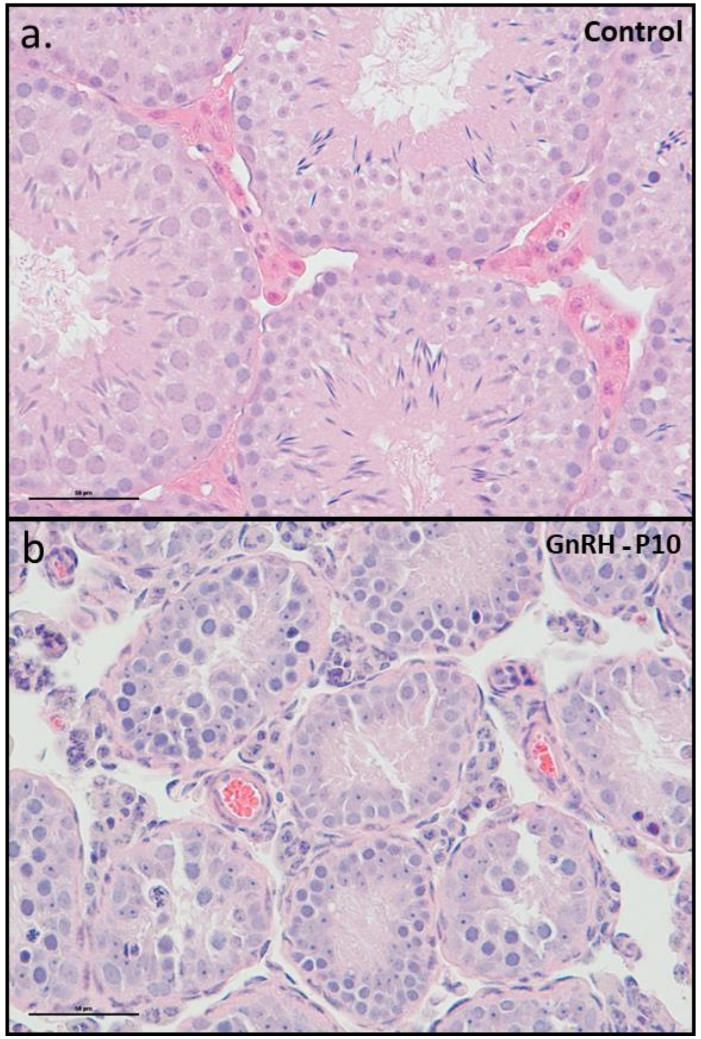


**Supplementary Figure 4.** Testis degeneration in cGnRH-P10 immunized mice. **(a)** H&E stained cross-section from PBS-immunized mice. Normal tissue architecture includes interstitial Leydig cells surrounding seminiferous tubules which contain subcapsular Sertoli cells and orderly arranged layers of maturing germ cells and spermatozoa. **(b)** Representative cross-section from cGnRH-P10 immunized mice. Presumptive interstitial Leydig cells appear clustered with fragmented nuclei. Tubules lined with Sertoli cells are significantly reduced in size, contain degenerating germ cells and are devoid of spermatids and spermatozoa. The bars denote a length of 50 uM.

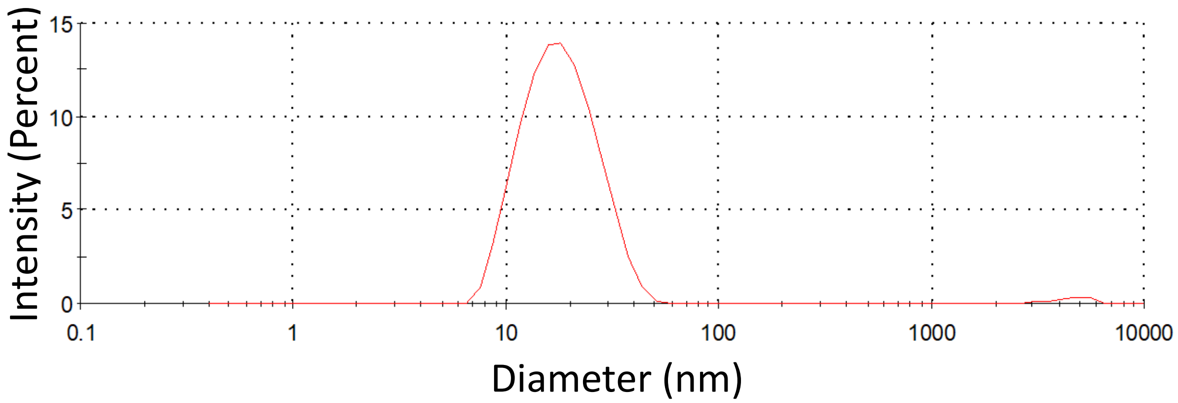


**Supplementary Figure 5.** DLS analysis of P10 modified with the mCε3 IgE epitope at its C-terminus.

a.

Ac-S**TQ**NA**ID**E**IT**N**KV**N-IKKIEKR-IKKIEKR-
abcdefgabcdefg-abcdefg

b.

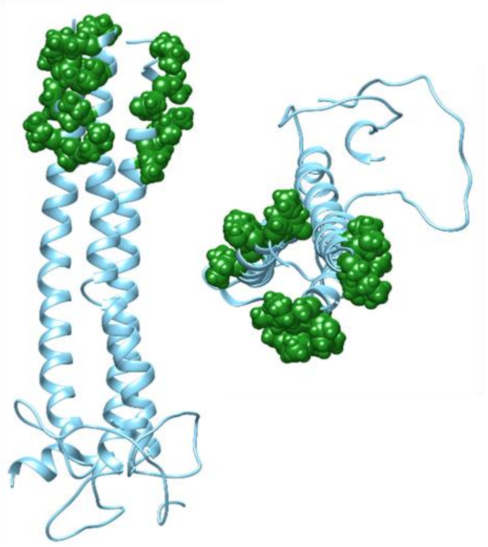

c.

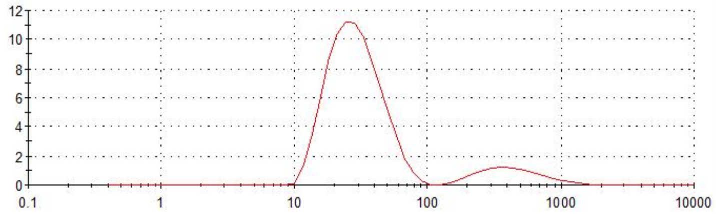


**Supplementary Figure 6.** Design of HxA$^{H1}$-P10. **(a)** Primary sequence of the N-terminal junction containing the HxA epitope from H1N1 virus followed by the first two heptads of the P10 self-assembly domain. The alignment of the HxA residues with the heptad register (red) predicts that the CR9114 contact residues (bold) are largely positioned in outward-facing heptad positions (b/c/f) to facilitate B cell recognition. **(b)** Molecular modeling of the trimer formed by HxA$^{H1}$-P10. CR9114 contact residues critical for antibody recognition are depicted in green. **(c)** DLS analysis of HxA$^{H1}$-P10.

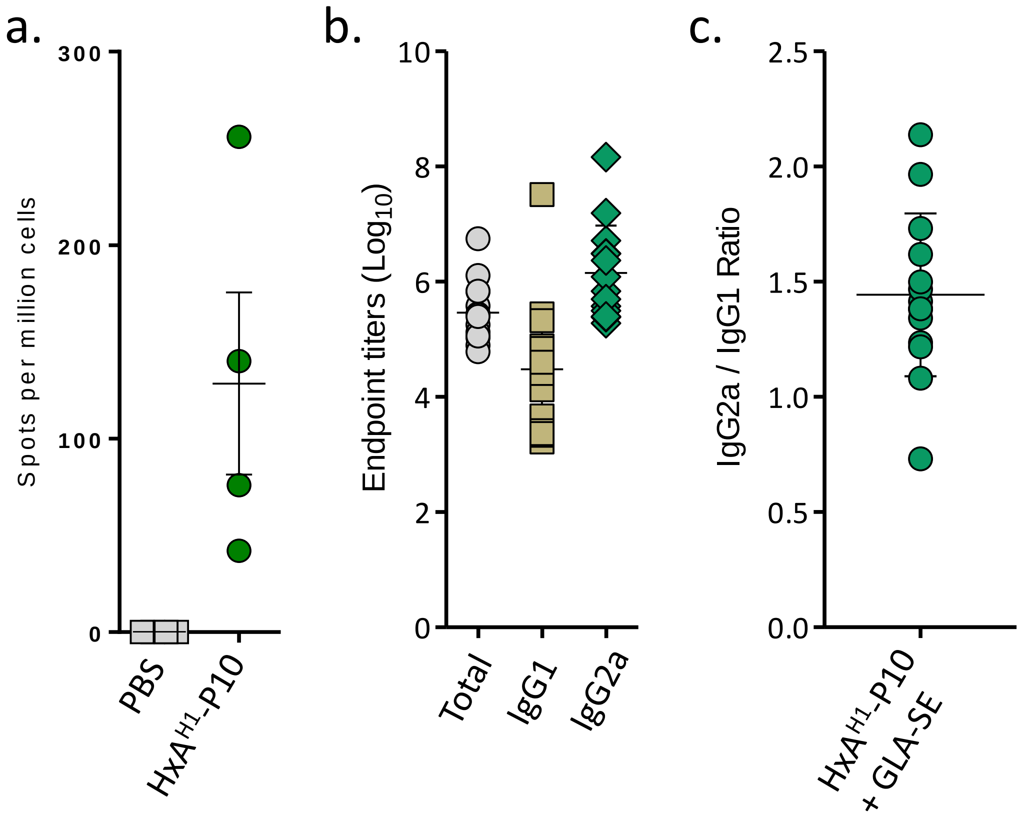


**Supplementary Figure 7.** HxA$^{H1}$-P10 induces strong CD4$^{+}$ T cell activity. CD-1 male mice (n=15) were immunized (d0, d21) with 10 µg of HxA$^{H1}$-P10 + GLA-SE or with PBS control. **(a)** Splenocytes (n=4) were harvested from group on d28 for T cell analysis. Cells were stimulated with the TCE dimer of P10 and then assayed for activity by IFN-γ ELISPOT. **(b)** Antisera from d35 was assayed by ELISA for titers to recombinant H1. Titers of total IgG, IgG1, and IgG2a are indicated. **(c)** The ratio between IgG2a and IgG1 isotypes. Values greater than 1 are indicative of a Th1 response, which is induced by the TLR4 agonist GLA.[22] T cell and isotype analysis studies were performed once.
